# Sleep-Disordered Breathing and Chronic Respiratory Infections: A Narrative Review in Adult and Pediatric Population

**DOI:** 10.3390/ijms24065504

**Published:** 2023-03-13

**Authors:** Paola Faverio, Umberto Zanini, Anna Monzani, Gianfranco Parati, Fabrizio Luppi, Carolina Lombardi, Elisa Perger

**Affiliations:** 1 UOC Pneumologia, Fondazione IRCCS San Gerardo dei Tintori, 20900 Monza, Italy; paola.faverio@unimib.it (P.F.); u.zanini@campus.unimib.it (U.Z.);; 2 School of Medicine and Surgery, University of Milano Bicocca, 20900 Monza, Italy; 3 Istituto Auxologico Italiano, IRCCS, Sleep Disorders Center & Department of Cardiovascular, Neural and Metabolic Sciences, San Luca Hospital, 20149 Milan, Italy

**Keywords:** sleep disordered breathing, obstructive sleep apnea, chronic respiratory infections, bronchiectasis, cystic fibrosis, non-tuberculous mycobacteria, tuberculosis

## Abstract

Sleep-disordered breathing (SDB) comprises different diseases characterized by abnormal respiratory patterns during sleep including obstructive sleep apnea. SDB prevalence and impact in patients with chronic respiratory infections have been only marginally studied. The purpose of this narrative review is to report the prevalence and impact of SDB in chronic respiratory infections, including cystic fibrosis (CF), bronchiectasis and mycobacterial infections, and explore the possible pathophysiological mechanisms. Common pathophysiological mechanisms, underlying SDB onset in all chronic respiratory infections, include inflammation, which plays a central role, chronic nocturnal cough and pain, excessive production of mucous plugs, presence of obstructive and/or restrictive ventilatory impairment, upper airways involvement, and comorbidities, such as alteration of nutritional status. SDB may affect about 50% of patients with bronchiectasis. The severity of the disease, e.g., patients colonized with P. aeruginosa and frequent exacerbators, as well as comorbidities, such as chronic obstructive pulmonary disease and primary ciliary dyskinesia, may impact SDB onset. SDB may also frequently complicate the clinical course of both children and adults with CF, impacting the quality of life and disease prognosis, suggesting that their routine assessment should be incorporated into the clinical evaluation of patients from the first stages of the disease regardless of suggestive symptoms, in order to avoid late diagnosis. Finally, although the prevalence of SDB in patients with mycobacterial infections is uncertain, extrapulmonary manifestations, particularly nasopharyngeal locations, and concomitant symptoms, such as body pain and depression, may act as atypical predisposing factors for their development.

## 1. Introduction

Sleep-disordered breathing (SDB) comprises different diseases characterized by abnormal respiratory patterns during sleep and is often associated with cardio-pulmonary comorbidities such as chronic heart failure, pulmonary embolism and pulmonary hypertension [1,2,3]. The main causes of SDB include obstructive sleep apnea (OSA), sleep hypoventilation, central sleep apnea (CSA) and sleep-related hypoxemia. OSA is the most common sleep disorder and it affects one-third of the population aged between 30 and 70 years in Europe [4], including a huge number of people who have not received a diagnosis yet [4,5,6,7].

Chronic respiratory infections may include difficult-to-treat infections or infections that require long-term antibiotic therapy, such as tuberculosis (TB) and non-tuberculous mycobacterial pulmonary disease (NTM-PD), and also structural lung diseases that predispose to chronic bacterial colonization, such as bronchiectasis and cystic fibrosis (CF). CF is a multisystem disease whose main respiratory manifestations include chronic bacterial colonization, productive cough, progressive bronchial distortion and remodeling leading to bronchiectasis, haemoptysis, atelectasis and pneumothorax. Progressive pulmonary failure continues to be the major cause of morbidity and mortality [8]. Bronchiectasis is a condition characterized by the permanent dilation of bronchi with the destruction of elastic and muscular components of their walls. Although they are primarily a structural lung disease, we discuss bronchiectasis among chronic respiratory infections because they may frequently cause a vicious circle from impaired muco-ciliary clearance with excessive production of mucous plugs leading to chronic airway infections and inflammation with continuous airways remodeling [9]. Mycobacterial infections, including TB and NTM-PD, typically affect patients with structural lung diseases, such as bronchiectasis, patients with risk factors for immunosuppression, and, in the case of TB, patients living in highly endemic countries.

SDB has been extensively evaluated in patients with other chronic obstructive airway diseases, such as chronic obstructive pulmonary disease (COPD) and asthma, but only marginally studied in patients with chronic respiratory infections such as bronchiectasis and mycobacterial infections.

Multiple causes may lay behind this lack of knowledge; first of all, chronic respiratory infections, including NTM-PD, and bronchiectasis have long been considered orphan diseases and, therefore, research on these topics has not flourished until recent years. Secondly, the main and most studied risk factors for SDB, such as obesity, are not so common in patients with chronic respiratory infections, hiding possible concomitant diseases. Thirdly, the typical symptoms that may lead to SDB diagnosis, e.g., excessive daytime sleepiness, snoring and nocturia, are not commonly reported by patients with chronic respiratory infections.

This review aims to evaluate the current evidence on the prevalence and impact of SDB in chronic pulmonary infectious diseases, including bronchiectasis, CF, TB and NTM-PD.

## 2. Material and Methods

A search of relevant medical literature in the English language was conducted in Medline/PubMed and EMBASE databases including observational, interventional studies and reviews on both adults and children through January 2023. Keywords used to perform the research are reported in Table 1. Editorials, narratives, conference abstracts and pre-print publications were excluded. All studies regarding chronic respiratory infectious diseases, the objective of this review (bronchiectasis, CF and mycobacterial infections) were included. Studies reporting only on other chronic respiratory diseases whose pathogenesis is not directly related to infections, such as COPD and asthma, were excluded. Relevant abstracts and articles were searched and screened independently by three authors (PF, UZ and EP) and, when there was a discrepancy between the authors, the articles were collectively discussed and analyzed for relevance, strengths and limitations. The most relevant literature regarding the three main topics of this review (bronchiectasis, CF and mycobacterial infections) is summarized in Table 2. We included articles in Table 2 (four on CF, two on bronchiectasis and one on mycobacterial infection); three were prospective and four were retrospective observational studies.

## 3. Sleep-Disordered Breathing and Chronic Respiratory Infections: Pathophysiology and Underlying Mechanisms

Sleep-related hypoventilation is common in neuromuscular diseases and chest wall disorders due to the physiologic reduction in ventilation during sleep together with the progressive chronic challenges imposed by the underlying diseases [17,18]. Hypoventilation is first seen during rapid eye movement (REM) sleep before progressing to non-REM sleep and wakefulness [19]. Clinical presentation is nonspecific and daytime respiratory function measures poorly predict nocturnal hypoventilation. The concomitant narrowing of the upper airway induced by apneas may contribute to worsening nocturnal hypoxemia. Central events derive from the instability of the breathing pattern caused by high loop gain, which may, on the other hand, also provoke obstructive events [20,21]. OSA is characterized by a partial or complete collapse of the upper airway during sleep, resulting in reduction or cessation of airflow despite the increased respiratory effort. The repetitive collapse of the pharyngeal airway during sleep leads to impaired gas exchange and intermittent oxygen desaturations that result in arousal from sleep. Intermittent and sustained nocturnal hypoxemia, particularly with concomitant hypercapnia, typical of OSA, CSA and sleep hypoventilation activates the sympathetic nervous system, being the major contributor to cardiovascular comorbidity of SDB [22]. These surges in sympathetic activity also result in ―the release of inflammatory mediators, lipolysis, and worsened insulin resistance, especially in OSA [23].

**Figure 1:**
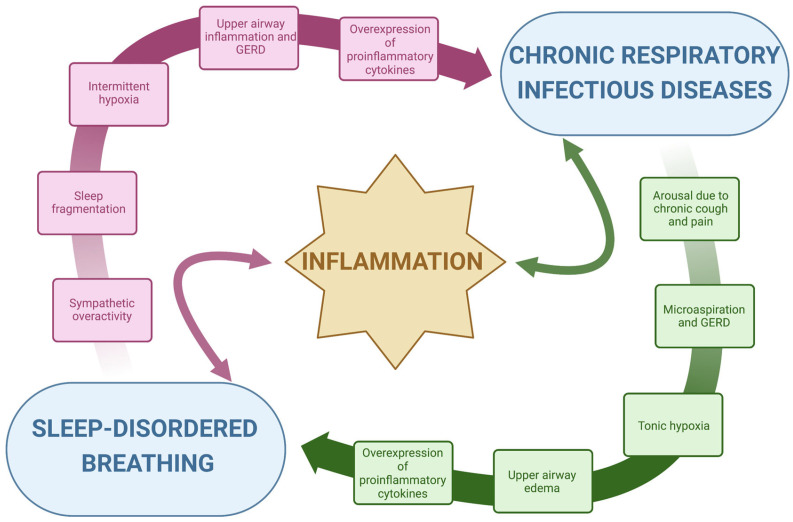
Inflammation cross-talk between chronic respiratory infectious diseases and sleep-disordered breathing.

Mechanisms that may justify the coexistence of SDB, especially OSA, and chronic respiratory infections leading to bronchiectasis are many. In particular, the synergic increases in proinflammatory stimuli and anatomical alterations of the upper airway might be the subjects of a cross-talk mechanism responsible for a bidirectional worsening of the two diseases, Figure 1. An over-sensitive ventilatory control system—or loop gain―and altered arousal threshold represent a predisposing condition for SDB in chronic infectious diseases [24,25,26,27].

A. Inflammation: The repetitive collapse of the pharyngeal airway characteristic of OSA leads to intermittent oxygen desaturation, sleep fragmentation and the consequent activation of the sympathetic nervous system, which is the major contributor to the release of systemic inflammatory mediators, Figure 1 [28,29]. Thus, intermittent hypoxia has been largely linked to major pro-inflammatory cytokines, such as tumor necrosis factor α (TNF-α) and interleukin 6 (IL-6), which constitute the classical prototypes of the large spectrum of systemic inflammation [29]. The subsequent cytokine-mediated inflammatory cascade, coupled with mechanical lung injury, damages the lungs and may worsen several conditions, including chronic respiratory infections [30]. Oxidative stress, such as the structural and functional alteration induced by reactive oxygen species, in response to chronic and intermittent hypoxia, is associated with airway damage [31,32]. In bronchiectasis, chronic bronchial infection and inflammation interact with each other and are responsible for progressive lung damage [33]. Oxidative stress and hypoxia in the airway are induced by the consumption of nutrients by inflammatory cells and bacteria and by a reduced supply of oxygenated blood to damaged lung segments [32]. The overexpression of pro-inflammatory cytokines due to SDB, especially in OSA, might accelerate this process precipitating the evolution of chronic respiratory diseases. On the other hand, the persistent inflammation induced by hypoxia, oxidative stress and chronic infections might predispose to SDB by increasing local phlogosis, gastroesophageal reflux disease (GERD) and, thus, upper airway edema. Upper airway inflammation and edema might increase pharyngeal collapsibility, as it will be described subsequentially. Given these premises, inflammation represents the subject of a bidirectional link between SDB and chronic respiratory infectious diseases, Figure 1. The disease-related chronic overexpression of inflammatory cytokines is enhanced in a vicious circle, with a consequent potential worsening of general clinical conditions.
B. Anatomy, upper airway edema and local inflammation: The occurrence of upper airway obstruction during sleep reflects an interplay between the removal of the wakefulness drive (which helps to maintain airway patency) and an individual anatomical predisposition with susceptibility to collapse. Pharyngeal muscle relaxation during sleep and lack of sufficient reactivation are key primary pathophysiological events leading to OSA [34]. Interstitial fluid accumulation in the upper part of the body during the night decreases the pharyngeal size and increases pharyngeal resistance and upper airway collapsibility in predisposed individuals [35,36]. A narrow upper airway importantly contributes to the development of OSA, typically worsened by a fat deposit in the parapharyngeal fat pads and pharyngeal muscles, obesity being one of the major risk factors for OSA, or by edema of the upper airway induced by local acute or chronic inflammation [28,37]. In patients with OSA, upper airway tissue is characterized by subepithelial edema and excessive inflammatory cell infiltration [38,39]. Chronic respiratory infectious diseases are characterized by chronic airway inflammation, which also involves the upper airways, GERD and microaspirations, especially at night time [40,41,42]. Acid regurgitation in the upper airway might contribute to the further narrowing of the pharyngeal region by local inflammation. In turn, OSA swings in intrathoracic pressure during apneas increase the pressure gradient between the esophagus and the stomach displacing the gastric contents into the esophagus, determining GERD. This produces a further pharyngeal spasm in patients with OSA and might decrease pharyngeal dilator muscle responsiveness by reducing specific receptors’ sensibility. GERD can also lead to bronchoconstriction or coughing in patients with lung diseases, by causing microaspiration. Additionally, OSA can also affect airway immunity leading to an increased propensity for respiratory tract infection-mediated exacerbations that can progress underlying chronic airway disease [43,44]. Indeed, the presence of upper airway symptoms was shown to increase disease duration and the exacerbation rate in patients with bronchiectasis [45]. Local upper airway inflammation related to chronic respiratory infections might therefore increase pharyngeal collapsibility, inducing pharyngeal narrowing in predisposed individuals.
C. Ventilatory drive instability and loop gain: respiration regulatory disturbances are essential in SDB pathogenesis, as respiratory control plays an essential role in maintaining stable respiration during sleep in healthy humans. The respiratory control system sensitivity is modulated through a negative feedback mechanism called loop gain [46,47,48]. A high loop gain is a marker of breathing instability and determines an exaggerated increase in ventilation in response to minimal changes in blood gas tension. Changes in respiration consequent to obstructive events (reduction in ventilation) or to arousals (increase in ventilation) evoke an exaggerated respiratory response when the loop gain of the subject is high. This response becomes a disturbance itself and will propagate breathing instability, and thus apneas and hypopneas, in predisposed individuals [49]. The cardinal symptom of chronic respiratory infections is chronic productive cough [50,51,52]. Nocturnal cough arouses the subject determining a sudden increase in respiratory rate and carbon dioxide changes. Accordingly, with the loop gain of the subject, the ventilatory drive may induce an increased response that will propagate respiratory instability and respiratory events in predisposed individuals [24]. Given these premises, cough and pain might be stimuli for SDB propagation in subjects with a high loop gain.
D. Arousals and arousal threshold: Arousals contribute to sleep fragmentation and poor sleep quality in subjects with SDB and chronic respiratory diseases and indirectly worsen the predisposition to develop sleep apnea. As mentioned above, recurrent abrupt arousals during sleep may contribute to the exaggerated post-event ventilatory response, reiterating respiratory instability and, thus, SDB [22,53]. The respiratory arousal threshold is the level of inspiratory mechanical effort required to wake up an individual in response to the narrowing of the upper airway during sleep. Although it has been postulated that a low arousal threshold may contribute to the development of OSA in predisposed subjects [24,54], delaying arousals in subjects with poor pharyngeal muscle responsiveness would increase the risk of severe overnight hypoxemia. Sustained isocapnic hypoxia increases the respiratory arousal threshold [55]. Increased arousal threshold together with sustained nocturnal hypoxemia may further impair the normal defense mechanisms that operate to minimize the result of abnormal breathing and gas exchange during sleep. This may have implications for disorders characterized by sustained nocturnal hypoxia, such as chronic respiratory infections, worsening the baseline hypoxic condition.

**Figure 2:**
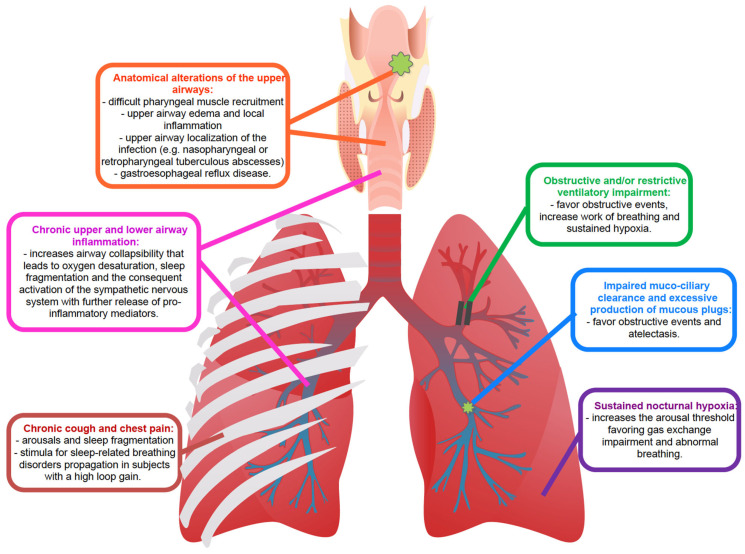
Summary of the main pathophysiological mechanisms causing SDB in chronic respiratory infections.

The main pathophysiological mechanisms causing SDB in chronic respiratory infections are summarized in Figure 2.

## 4. Sleep-Disordered Breathing and Bronchiectasis

Bronchiectasis is an anatomical alteration with a permanent enlargement of part of the airways. Many different causes may contribute to the development of bronchiectasis, including prior pulmonary infections, such as tuberculosis, autoimmune diseases and impaired host defenses, such as primary ciliary dyskinesia (PCD) or immunodeficiencies. CF-associated bronchiectasis for this review will be addressed in a specific paragraph.

It has been shown that patients with bronchiectasis have multiple risk factors for SDB, such as chronic inflammation and ventilatory impairment due to obstructive and/or restrictive patterns and chronic secretions, caused by impaired muco-ciliary clearance and by excessive production of mucous plugs [56,57]. Only a few studies have thoroughly investigated the prevalence of SDB, particularly OSA, in patients with bronchiectasis [10,11]. Two studies conducted in Brazil and Turkey reported the results of polysomnography (PSG) in 49 and 43 patients with bronchiectasis, respectively. Prevalence of OSA ranged between 41% and 56% and, in more than half cases in both studies, it was of mild severity (apnea-hypopnea index (AHI) 5–15). Risk factors for the presence of OSA were older age, male gender, larger neck circumference and *P. aeruginosa* colonization [10,11]. Contrary to what was expected, body mass index (BMI), pulmonary functional parameters and the number of prior exacerbations did not differ between patients with or without OSA. Smoking history was not evaluated in these studies, probably because pathophysiological mechanisms for OSA in patients with bronchiectasis may at least in part be different from those of the general population. Recognized markers of bronchiectasis severity, such as the number of exacerbations per year ≥ 3, were associated with longer snoring periods, worse oxygen saturation (SpO_2_) nadir and higher total sleep time with SpO_2_ < 90% [10]. To our knowledge, literature is lacking regarding the impact of treating SDB in these patients and whether airway-clearance techniques and pulmonary rehabilitation may have a positive effect on OSA. Regarding diagnosis, typical symptoms suggestive of OSA may not be present in patients with bronchiectasis. In the study by Faria Junior et al., daytime levels of sleepiness evaluated using the Epworth sleepiness scale (ESS) did not differ between patients with and without OSA [11]. Similarly, comparing subjects with bronchiectasis with and without OSA, Borekci et al., did not observe any difference regarding excessive daytime sleepiness and snoring [10]. These findings suggest that patients with bronchiectasis may require screening for OSA regardless of typical risk factors and symptoms.

The impact of sleep disturbances on the quality of life in both children and adults with bronchiectasis was also investigated through questionnaires [58,59]. Gao et al., applied the Pittsburgh Sleep Quality Index (PSQI), ESS and St. George Respiratory Questionnaire in 144 adults with bronchiectasis and observed that they had a higher prevalence of sleep disturbances (based on the PSQI score > 5), but no difference in daytime sleepiness (based on the ESS score > 10) compared to healthy subjects [58]. Furthermore, compared to patients without sleep disturbances, those with SDB according to the questionnaire response had a more impaired quality of life. Erdem et al., collected the PSQI and the Pediatric Sleep Questionnaire in 54 children with bronchiectasis and age-matched controls [59]. The prevalence of SDB and poor sleep quality was higher in patients with bronchiectasis compared to controls (22 vs. 9% and 37 vs. 17%, respectively). Moreover, patients with chronic symptoms such as sputum production, snoring and wheezing, and those with worse high-resolution computed tomography involvement had poorer sleep scores [59]. Both the studies by Gao et al., and Erdem et al., showed that sleep quality was impaired in a non-negligible percentage of patients with bronchiectasis, regardless of age [59]. However, neither of the two studies performed PSG to definitively confirm the presence of SDB.

Bronchiectasis may also complicate other diseases, such as COPD, and increase the prevalence of OSA. A recent observational study by Yang et al., studied the prevalence of bronchiectasis, with chest computed tomography, and the presence of OSA, through nocturnal PSG, in 124 consecutive patients with COPD, mostly of severe entity [60]. Bronchiectasis were significantly more frequent in patients with COPD-OSA overlap syndrome than in those with COPD without OSA (43 vs. 19%) Furthermore, the co-presence of bronchiectasis and OSA in COPD patients was related to more severe nocturnal hypoxia, evaluated through the percentage of time spent with SpO_2_ below 90%, and higher systemic inflammation, according to through C-reactive protein levels [60].

Finally, the prevalence of SDB has also been studied in patients with primary ciliary dyskinesia (PCD) a possible cause of both bronchiectasis and chronic rhinosinusitis. Two studies conducted in Italy and Turkey compared children with PCD (16 in the Italian and 29 in the Turkish study) with healthy controls and found a higher rate of SDB and poorer sleep quality in those with PCD [61,62]. In particular, the bronchiectasis severity score was negatively associated with SpO_2_ in both studies.

In conclusion, according to the limited data available to date, SDB may affect about 50% of patients with bronchiectasis, although they may have been under-reported in many studies. The severity of the disease, e.g., patients colonized with *P. aeruginosa* and frequent exacerbators, as well as comorbidities, such as COPD and PCD, may impact SDB onset.

## 5. Sleep-Disordered Breathing and Cystic Fibrosis

SDB has been extensively studied in children and adolescents with CF [63]. Differently, studies on adults affected by CF have been limited due to the small sample sizes and lack of diversity [64]. The main sleep abnormalities observed in both adult and pediatric patients with CF range from nocturnal hypoxemia and/or hypercapnia to increased respiratory rate during sleep and OSA [65]. The pathogenesis of SDB in CF resembles that of bronchiectasis, where chronic infection and inflammation together with accumulation of mucous plugs play a pivotal role.

Different studies have shown that SDB, including OSA, may occur in patients with CF even before daytime clinical manifestations, suggesting that early screening is important in this population [65,66]. SDB and nocturnal episodes of oxygen desaturations have also been described in infants with CF under 3 years of age with mild airway inflammation (rhinitis, cough, red throat) [67].

Furthermore, different from patients with bronchiectasis and mycobacterial infections, patients with CF often manifest poor sleep quality with frequent awakenings and daytime sleepiness, and such disturbances are more frequent with the progression of the disease [68]. Despite this, SDB prevalence is still under-recognized in this population of patients and may impact disease outcomes [69]. As an example, in CF patients with a severe disease complicated by pulmonary hypertension and right ventricular failure, chronic nocturnal hypoxia secondary to untreated SDB may worsen the disease leading to a poor prognosis [70].

Multiple studies have explored the possibility that markers of CF severity, including lung function, may predict SDB presence and severity with conflicting results [13,15,16]. On one hand, Shakkottai et al., in a retrospective analysis of patients with CF did not find any association between OSA detected by PSG and clinical signs of CF severity, suggesting the need for routine PSG to screen for sleep disorders [13,15]. On the other hand, Lumertz et al., found that forced expiratory volume in the 1st second, a marker of obstructive impairment at spirometry, was directly correlated with mean sleep SpO_2_ and negatively correlated with sleep peak end-tidal carbon dioxide [16].

A recent meta-analysis by Pedrada De Sousa et al., included 6 studies to investigate the prevalence of OSA in children and adolescents with CF and preserved or mildly impaired lung function. The pooled prevalence, considering an obstructive AHI > 2 per hour, was 52%, regardless of lung function impairment [71].

The available literature on adult patients with CF reported a wide range of OSA prevalence. In a cross-sectional study, Perin et al., compared 51 stable adults with CF to 25 age-matched controls to evaluate sleep parameters and to determine predictors of nocturnal desaturation. In this study, the estimated prevalence of OSA in adults with CF was 3.9%, which is less than the general population [64,72]. Similar results were reported by Milross et al., while Welsner et al., reported a higher prevalence (40%) [73,74]. These conflicting results suggest that the prevalence of OSA is still unknown and may be greatly underestimated [75,76].

In contrast, results about the incidence of nocturnal hypoxia in adult patients with CF are more consistent. Perin et al., showed that the incidence of nocturnal desaturation was more common in adult patients with CF than controls (29.4% vs. 0%; *p*  <  0.001) and the best predictor for sleep desaturation was the awake resting SpO_2_ [64]. The study by Milross et al., reported a similar incidence (25%) of nocturnal desaturation, also describing a correlation between the occurrence of nocturnal hypoxia and the severity of lung involvement in CF. [70].

SDB in children and adults with CF also deeply affects daytime function and quality of life, in fact, the presence and severity of SDB have been inversely associated with exercise capacity and daily physical activity levels [14,64,77]. Moreover, a direct link was detected between nocturnal hypoxemia and exercise intolerance, and between sleep architecture disorders and sedentary physical activity levels [14].

Furthermore, not only respiratory parameters such as lung function but also other systemic parameters, such as nutritional status (overweight/obesity), have been described as major determinants of OSA [15,78]. The development of OSA in patients with CF was not only associated with pulmonary manifestations of the disease but also with the presence of upper airway obstruction caused by chronic rhinosinusitis, nasal polyposis and tonsillar hypertrophy [79].

Supplemental oxygen and non-invasive ventilation (NIV) have been applied in both adults and children with CF and SDB and are considered to be effective in the short term. However, long-term data are still needed [65,80].

To prevent hypoventilation and nocturnal hypoxia, Young et al., examined the role of NIV in adults with CF [81]. This study compared the use of nocturnal NIV to low-flow oxygen and to room air (as a placebo) over 6 weeks in a randomized, placebo-controlled, crossover trial. In comparison to room air, NIV improved chest symptoms (evaluated with the CF Quality of Life Questionnaire), exertional dyspnea and peak exercise capacity in patients with stable CF and awake hypercapnia [81]. Similarly to the aforementioned study, Wadsworth et al., showed the improvement of lung function and the attenuation of hypercapnia in adults with CF using nocturnal NIV [82].

In conclusion, routine checks for SDB should be incorporated into the clinical evaluation of patients with CF from the first stages of the disease. Suggestive symptoms, such as daytime sleepiness, and lung disease severity may lack sensitivity and lead to late diagnosis.

## 6. Sleep-Disordered Breathing and Mycobacterial Infections

TB is an infectious disease caused by Mycobacterium Tuberculosis Complex (e.g., *M. tuberculosis*, *M. Africanum* and *M. bovis*). It mainly involves the lungs, but in one-fifth of cases can also present with extrapulmonary manifestations. It was recently estimated that about 22% of the world population is infected, with a latent or active form, with *M. tuberculosis* [83]. Inflammation and proinflammatory mediators strongly impact the severity and progression of the disease [84,85].

Non-tuberculous mycobacterial (NTM) infections are caused by mycobacterial species other than Mycobacterium Tuberculosis Complex. NTM infection mainly involves the lung with rare extrapulmonary manifestations. The incidence worldwide is lower than TB, but it is increasing, particularly in high-income countries [86]. In fact, in the USA, from 2008 to 2015, the annual incidence of NTM-PD increased from 3.1 to 4.7 per 100,000 person-years [87]. Treatment of NTM infection is particularly challenging with high rates of NTM isolation relapse or reinfection (up to 50% of patients who completed treatment), despite an initially successful antibiotic treatment [88]. Furthermore, specific antimicrobial therapy requires at least a 3-to-4 drug regimen administered for at least 15 to 18 months [88]. These above-mentioned characteristics make the NTM-PD effectively a chronic infection.

The prevalence of SDB in mycobacterial infections, as well as the mutual impact of SDB on mycobacterial infection and vice versa, have only scarcely been investigated and the few studies available show discordant results [89,90]. In a recent review, Devassy et al., report that poor sleep quality and restless leg syndrome were higher in patients with TB compared to the general population [89]. Authors also speculated that symptoms associated with SDB may exacerbate chronic infections like TB by impairing immune regulation [89]. A population-based study by Lee et al., evaluated the incidence of TB in patients with OSA using data from the Taiwan National Health Insurance database [90]. They compared the incidence of TB between 6135 patients with OSA and 184,050 control subjects without OSA [90]. The prevalence of TB was significantly higher in the control group (0.56%) than in patients with OSA (0.33%), suggesting that the incidence of TB in patients with OSA is lower than in those without.

Extrapulmonary manifestations of TB may favor SDB onset when localized in the upper airway region. OSA was observed in two patients with nasopharyngeal TB and tuberculous retropharyngeal abscess [91,92]. Considering that nasopharyngeal TB affects 1.9% of patients with pulmonary TB, OSA might complicate the clinical course of these patients. Although rare, cervical masses were reported in 59% of patients with nasopharyngeal tuberculosis [93]. The obstruction of the nasopharynx increases the calibre and resistance of the upper airway, lowering the airflow and eventually causing snoring and OSA.

SDB was also evaluated in patients with sequelae of pulmonary TB, including pleural thickening, prior pulmonary resection and/or atelectasis, fibrosis, bronchiectasis, cavity formation and compensatory emphysematous changes [94]. In these cases, the development of SDB is not directly related to active infection or inflammation, but to their consequences leading to a restrictive ventilatory impairment and, in some cases, chest wall diseases. Sakuma et al., in 1997 reported the characteristics of sleep oxygen desaturations in 38 patients with TB sequelae in comparison to 40 patients with COPD [94]. The baseline nocturnal SpO_2_ was similar between the two groups, however, the lowest sleep SpO_2_ was more profound in the TB sequelae group. Most of the patients in the study were treated before the 1960s with thoracoplasty because at the time there was hardly any effective antimicrobial regimen for TB [94]. Therefore, possible reasons for the deeper nocturnal desaturations were mechanical disadvantages (e.g., pulmonary fibrosis and loss of lung volume) secondary to thoracoplasty. However, nowadays the surgical approach for TB is rare since the first-line therapy for TB is multiple antibiotic regimens with lower possibilities of developing permanent structural sequelae [94].

Finally, only one study investigated the presence of SDB in NTM-PD in association with other symptoms, such as depression [95]. In a cross-sectional retrospective study, Matsumura et al., analyzed the prevalence of depressive symptoms and the factors that influenced their development in 114 patients with NTM-PD [95]. The authors found that 32.5% of patients reported depressive symptoms, in which an important role was played by disease duration and sleep disturbances.

In conclusion, several speculations have been made about the possible association between SDB and mycobacterial infections. However, the few studies available are inconclusive regarding the prevalence of SDB. Furthermore, the possible role of extrapulmonary manifestations of the infection (e.g., nasopharyngeal locations) and concomitant symptoms, such as depression, have only been studied and reported anecdotally.

Given that this topic is not supported by high-quality literature and that the few studies available did not implement in their methodology complete sleep studies, we cannot draw any conclusion other than to indicate the need for high-quality prospective studies.

## 7. Consequences of Coexistence of SDB and Chronic Respiratory Infectious Diseases

A. Misdiagnosis: obesity is an important risk factor for OSA since OSA incidence is directly related to increased BMI [96]. Fat deposits in the upper respiratory tract narrow the airway, leading initially to snoring and, subsequently, resulting in sleep apnea with weight gain and worsening of the obstruction. Patients with chronic respiratory infections are generally normal weight or underweight due to persistent chronic infection and inflammation. The absence of snoring, as a reported symptom, and the absence of a typical OSA patient phenotype might reduce the suspicion of clinicians leading to the underestimation of SDB. Moreover, unexplained chronic cough has been reported in patients who snore and who have SDB and, as previously explained, it is also one of the peculiar symptoms of CF, NTM-PD and bronchiectasis [97]. In certain cases, chronic cough can be the sole manifestation of OSA, when specifically investigated by sleep clinicians during a visit [98]. In patients known for having respiratory diseases, such as bronchiectasis or chronic respiratory infections, cough might be explained by these underlying diseases leading to an underestimation of possible OSA symptoms and determining a misdiagnosis [15].
B. Hypoxia: hypoxia has deleterious effects on the cardiovascular system, the central nervous system and all the organs of the human body [99]. Many chronic respiratory diseases, including COPD, interstitial lung diseases and chronic respiratory infectious diseases, determine normobaric hypoxia based on different pathophysiological mechanisms [99]. Susceptible subjects with chronic respiratory comorbidities show a lower SpO_2_ than healthy subjects, especially during night-time. Lung infections, such as those mediated by mycobacteria, and bronchiectasis are both characterized by ventilation/perfusion mismatch due to regional lack of ventilation with consequent hypoxia [31]. In the airways of patients with CF, chronic hypoxia is also driven by impaired ventilation due to airway mucus obstruction [100]. As a complication of infections, atelectasis further reduces gas exchanges. The presence of SDB, particularly OSA and CSA, can further worsen tonic hypoxia by adding intermittent episodes of oxygen reduction [11,13,14,101,102]. Thus, CF subjects with SDB had lower SpO_2_ and each unit increase in AHI was associated with a decline in SpO_2_ nadir [13]. Oxidative stress and inflammatory pathways induced by intermittent hypoxia can be compounded by inflammation due to persistent infections, airway chronic damage and gas exchange alterations due to chronic lung diseases [44,103]. The high rate of underdiagnosed – and undertreated—OSA in conditions of chronic hypoxia such as in chronic respiratory infectious disease, might act as a cofactor in worsening patients’ nocturnal hypoxia and, thus, general clinical conditions. The overlap of the two diseases represents a risk factor itself for exacerbations and an increased susceptibility to worse respiratory outcomes [14,16].
C. Sleep fragmentation: cough and, secondarily, pain are hallmarks of chronic respiratory infections and when presenting at nighttime, are responsible for waking the patient and inducing sleep fragmentation. As a consequent mechanism, sleep fragmentation has effects on cognitive function, and alterations in the neuroendocrine, immune and inflammatory systems [104]. Sleep fragmentation and deterioration of sleep quality, typical of SDB, further complicate the fatigue and physical exhaustion often experienced by patients with chronic lung diseases [12]. Poor sleep quality will also impact the physiological beneficial effects on the immune system attributable to efficient sleep [105]. Moreover, poor sleep has been shown to increase the perception of pain [106]. Mori and coauthors reported worse pain experiences in subjects with poorer sleep quality [12]. OSA-related nocturnal hypoxemia, sleep fragmentation, and systemic inflammation impact pain perception by influencing the anti-nociceptive mechanism and aggravating both chronic and acute pain [107]. Worsening sleep fragmentation and the co-existence of SDB on top of a chronic respiratory infectious disease might increase systemic inflammation and pain perception aggravating the general clinical condition and patient’s quality of life [12,14,16,108].

## 8. Limitations

This narrative review has some limitations beyond those inherent in the narrative review, which is the weakest review type. First of all, since the literature on the prevalence and impact of SDB in chronic respiratory infectious diseases is very limited and heterogeneous it was impossible to conduct a methodologically superior review, such as a systematic review. Secondly, given the weak evidence available we specified that some speculations proposed by the authors of this review require stronger research to be confirmed. Thirdly, we did not collect and report information on several studies (i) identified from the research, (ii) screened, and (iii) excluded from the review and this may have introduced potential biases from incomplete reporting. Finally, we did not perform a formal assessment of the quality of the studies.

## 9. Conclusions

Chronic infectious respiratory diseases and SDB might coexist, aggravating the outcome and the subject’s quality of life due to local and systemic inflammation, intermittent hypoxia, sometimes superimposed to chronic hypoxia, and sleep fragmentation. Disrupted sleep and cough might worsen or induce OSA in susceptible patients by increasing upper airway edema, by determining ventilatory instability, or due to a potentially infectious disease localization in the upper airways, such as nasopharyngeal TB. Proinflammatory stimuli triggered by chronic respiratory diseases and enhanced by over-imposed SDB might worsen the prognosis and patient’s quality of life. Although, in the authors’ opinion, the cross-talk link between the diseases is evident, unfortunately, very few studies thoroughly evaluated this subject.

The limited literature available in this field might be related to the difficult identification of patients at risk of SDB with possible misdiagnosis caused by a lack of common known symptoms typical of OSA. For patients with chronic respiratory disorders, the available evidence suggests that the prompt recognition and treatment of SDB improves their quality of life and may also alter the course of the disease. Since the pathophysiology and the outcomes of the diseases are strictly related, there is now a need for epidemiologic and prognostic studies to better understand the prevalence, risk factors and impact of the superimposition of SDB on chronic respiratory infections. Research should focus first on the epidemiological distribution of SDB among patients with respiratory infectious diseases. Furthermore, the clinical impact of the coexistence of the diseases needs to be confirmed in observational and prognostic studies, also to confirm the pathophysiologic mechanisms underlying the diseases. Researchers also need to focus on the implication of interventions reducing SDB in populations affected by chronic lung infections. The results of these studies will help to drive clinicians to the best diagnostic and therapeutic approach for these patients. According to CF and bronchiectasis prevalence, this might be of particular relevance in the pediatric population.

## Figures and Tables

**Table 1:** Keywords used to perform the research.

Sleep-related breathing disorders (OR sleep quality OR obstructive sleep apnea OR sleep-disordered breathing) AND bronchiectasis (OR non-cystic fibrosis bronchiectasis);
Sleep-related breathing disorders (OR sleep quality OR obstructive sleep apnea OR sleep-disordered breathing) AND cystic fibrosis;
Sleep-related breathing disorders (OR sleep quality OR obstructive sleep apnea OR sleep-disordered breathing) AND tuberculosis (OR mycobacteria);
Sleep-related breathing disorders (OR sleep quality OR obstructive sleep apnea OR sleep-disordered breathing) AND non-tuberculous mycobacteria (OR non-tuberculous mycobacterial pulmonary disease OR mycobacteria other than tuberculosis OR atypical mycobacteria).

**Table 2:** Summary of the most relevant literature regarding the three main topics of this review (bronchiectasis, cystic fibrosis and mycobacterial infections).

Author- Journal-Year	Aim	Design	Inclusion/Exclusion Criteria	Study Groups	Outcome Measure	Main Results	Strengths/Limitations/Notes
Borekci S, Turk Thorac Journal, 2021 [10]	The objective is to investigate the frequency of OSA and related parameters in patients with NCFB.	Single center, prospective, observational study.	Inclusion criteria: - Diagnosis of bronchiectasis on HRCT - >18 years of age - Negative cystic fibrosis sweat test Exclusion criteria: - CF - Infectious attack in the last month - LTOT or NIV - Comorbidities that might be at risk for OSA	Including patients (n = 75), PSG performed (n = 45), and subjects enrolled (n = 43).	- Clinical, Demographic, and Anthropometric Features, including sputum colonization, bronchiectasis localization and type, number of lobes involved, and functional data) - Incidence of mild/moderate/severe OSA - Polysomnographic Parameters - Incidence of REM-dependent and position-dependent OSA	The frequency of OSA in patients with NCFB is 55.8% and increases with age. Investigating OSA using PSG is important in NCFB patients, especially at advanced ages.	Limitations: - Single center - Absence of a control group Strengths: - PSG for the diagnosis of OSA - Primary ciliary dyskinesia and CF exclusion
Faria Júnior NS, Plos One, 2017 [11]	The objective is to describe the physiological variables of sleep in patients with NCFB through PSG and to stratify these patients by the risk of OSA and excessive daytime sleepiness.	Two center, cross-sectional observational study.	Inclusion criteria: - Diagnosis of bronchiectasis based on HRCT - Age between 18 and 65 years - Use of a long-acting bronchodilator - Clinical stability for at least one month Exclusion criteria: - CF - History of smoking - Other lung diseases (e.g., chronic obstructive pulmonary disease or asthma)	Eligible patients (n = 418), Clinical evaluation (n = 50), Subjects analyzed (n = 49).	- Clinical, demographic, and anthropometric characteristics and comorbidities - Spirometric data - Polysomnographic physiological variables, Epworth Sleepiness Scale score and Berlin Questionnaire	Adult patients with clinically stable NCFB, especially those infected with PA, exhibit EDS and high prevalence of OSA, associated with considerable oxygen desaturation during sleep.	
Mori K, Medicine, 2021 [12]	The objective is to determine the prevalence and severity of body pain in patients with NTM-PD. The study also investigates the clinical indicators that contribute to pain.	Single center, retrospective cross-sectional study.	Inclusion criteria: - NTM-PD patients receiving pulmonary rehabilitation Exclusion criteria: - Clinical instability in the last three months - Missing laboratory data	Eligible patients (n = 180), Included and analyzed (n = 114). Divided into two groups: No pain group (n = 54), pain group (n = 60).	- Clinical, demographic, and anthropometric characteristics and comorbidities - Pulmonary function tests - Radiographic features - NTM species - Respiratory symptoms - Pain and pain-related medications - Body pain (score), mMRC (grade); ISWD (m); ISWD % pred (%); CES-D (score); CES-D (>15); PSQI (score); PSQI (>5); LCQ total (score)	Approximately 70% of patients with NTM-PD reported experiencing pain, and of these, over 1/3 report moderate to very severe pain. Factors predicting pain included the presence of depressive symptoms, poor sleep quality, and reduced exercise tolerance.	Limitations: - The sample was almost entirely female - No details about pain assessment
Shakkottai A, Sleep Medicine, 2020 [13]	Assess the frequency and severity of SDB in children and adults with and without CF (1:2), who were referred due to concerns for SDB.	A single-center, retrospective study.	Inclusion criteria: - CF patients with SBD - Not CF patients with SBD similar to CF patients Exclusion criteria: - Patients with chromosomal anomalies, chronic respiratory failure, neuromuscular conditions, upper airway abnormalities	CF group included 29 children and 23 adults; The non-CF group included 58 children and 46 adults.	- Baseline Demographics - CF-specific characteristics (FEV1, Brasfield score, sweat chloride, CF genetics) - Standard polysomnographic measures and nocturnal gas exchange (total sleep time, sleep-onset latency, REM sleep latency, sleep efficiency, WASO, arousal index, stage-shifts index, AHI, OAI, CAI, mean SpO_2_, minimum SpO_2_, percent total sleep time with low SpO_2_, duration of low SpO_2_, maximum CO_2_)	Subjects with vs. without CF had 3 times greater odds of moderate-severe SDB. Nocturnal SpO_2_ nadir was lower among CF vs. non-CF groups. For every 1-unit increase in AHI, the decline in minimum SpO_2_ was larger for subjects with vs. without CF. For every 1-unit increase in AHI, the magnitude of the decline in minimum SpO_2_ was larger for those with low vs. normal FEV1.	Limitations: - Retrospective study - Mild comorbidities - Matching the groups for demographic variables prevented an assessment of how these variables might affect differences in SDB between the CF and non-CF groups
Barbosa RRB, Pediatric Pulmonology, 2020 [14]	The objective is to evaluate the presence of SDB among children and adolescents with CF, attempting to identify associations with pulmonary function, nutritional status, days in hospital, and days taking antibiotics.	Single center, cross-sectional observational study.	Inclusion criteria: - CF patients from 6 to 18 years of age - Stable conditions and preserved cognitive function - Exclusion criteria: - Intercurrent conditions during the preceding 30 days - LTOT - The inability of the patient to understand or complete tests	Assessed for eligibility (n = 93), Invited to participate (n = 57), Included and analyzed (n = 31).	- Demographic, clinical and pulmonary function variables (e.g., chronic colonization, nasal polyposis, SK score, hospitalizations, antibiotic use, pulmonary function). - Main polysomnographic variables (including sleep latency, REM sleep latency, TST, NREM stages, REM sleep, WASO, arousal, arousal index, respiratory events, AHI, OAHI, sleep SpO_2_, NREM sleep desaturation index, SpO_2_ > 90%).	Children and adolescents with CF show SDB, including OSA (32.3%) and nocturnal hypoxemia (29%). Individuals with nocturnal hypoxemia had lower lung function, worse clinical scores, and higher morbidity. TST with SpO_2_ less than 90% was associated with the length of hospitalization.	Limitations: - Cross-sectional design - Unavailability of a capnograph for PSG - Absence of data on tonsil size and Mallampati score. - Use of home-based PSG - Small sample size
Shakkottai A, Pediatric Pulmonology, 2022 [15]	The objective is to identify demographic and CF-specific risk factors for OSA in a cohort of sleep-laboratory referredpatients with CF.	A single center, retrospective study.	Inclusion criteria: - CF patients who performed a PSG from January 2009 to October 2017 at Michigan Medicine Sleep Disorders Center Exclusion criteria: - LTOT - Patients without OSA but with other forms of SBD	Assessed for eligibility (n = 88), Included and analyzed (n = 74).	- General and CF-specific demographics (e.g., CFTR mutations, Brasfield score, tonsillar hypertrophy, nasal poliposis and chronic sinusitis) - Sleep duration, sleep fragmentation, and nocturnal gas-exchange variables	Key risk factors for OSA may differ between children and adults with CF: upper airway pathology appears important in children, overweight/obese or a crowded oropharynx in adults. Neither snoring, EDS, nor lung disease severity was associated with OSA.	Limitations: - Single center retrospective review - Small sample size Strengths: - Provide information that can be useful clinically for SDB’s screening
Lumertz MS, Sleep Science, 2019 [16]	Describe the frequency of SDB in pediatric CF patients and evaluate the associations between PSG respiratory parameters and Clinical information.	A single center, retrospective, cross-sectional study	Inclusion criteria: - CF patients who performed a PSG during the previous two years - Age between 2 and 20 years Exclusion criteria: - Nocturnal ventilatory support - Prior lung transplant - Unavailability of medical records - Irregular follow-up	Assessed for eligibility (n = 91), Included and analyzed (n = 16).	- Date of birth, sex, age of diagnosis - Anthropometric data and indicators of nutritional status - Comorbidities, treatment, number of exacerbations per year, Schwachman score and bacterial airway colonization - Lung functional data - PSG data	SDB was frequently observed in children with CF. There was an association between respiratory disease progression markers and sleep breathing parameters in children. Sleep studies appear to be an important tool for the assessment of the respiratory status.	Limitations: - Small sample size

Footnotes: AHI = Apnea-hypopnea index; BMI = Body mass index; CAI = central apnea index; CF = Cystic fibrosis; CO_2_ = carbon dioxide; EDS = Excessive daytime sleepiness; FEV1 = Forced expiratory volume in one second; HRCT = High-resolution computed tomography; LTOT = Long-term oxygen therapy; NCFB = Non-cystic fibrosis bronchiectasis; NIV = Non-invasive ventilation; NREM = non-rapid eye movement; NTM-PD = Nontuberculous mycobacterial pulmonary disease; OAHI = Obstructive apnea hypopnea index; OAI = Obstructive apnea index; OSA = Obstructive sleep apnea; OSAS = Obstructive sleep apnea syndrome; PA = Pseudomonas aeruginosa; PSG = Polysomnographies; REM = rapid eye movement sleep; SDB = sleep-disordered breathing; SK = Shwachman-Kulczycki; SpO_2_ = mean oxygen saturation; TST = Total sleep time; WASO = Wakefulness after sleep onset; mMRC = Medical Research Council dyspnea scale; ISWD = Incremental Shuttle Walk Test distance; ISWD % pred = Incremental Shuttle Walk Test distance percent predicted; CES-D = Center for Epidemiological Studies depression scale; PSQI = Pittsburgh Sleep Quality Index; LCQ = Leicester Cough Questionnaire.

## Data Availability

Not applicable.

## Abbreviations

AHI	apnea-hypopnea index
BMI	body mass index
CF	cystic fibrosis
COPD	chronic obstructive pulmonary disease
CSA	central sleep apnea
ESS	Epworth sleepiness scale
GERD	gastroesophageal reflux disease
NIV	non-invasive ventilation
NTM	non-tuberculous mycobacterial
NTM-PD	non-tuberculous mycobacterial pulmonary disease
OSA	obstructive sleep
PCD	primary ciliary dyskinesia
PSQI	Pittsburgh Sleep Quality Index
REM	rapid eye movement
SDB	sleep-disordered breathing
SpO_2_	oxygen saturation
TB	tuberculosis

## References

[1] García-Ortega A., Mañas E., López-Reyes R., Selma M.J., García-Sánchez A., Oscullo G., Jiménez D., Martínez-García M.Á. (2019). Obstructive Sleep Apnoea and Venous Thromboembolism: Pathophysiological Links and Clinical Implications. Eur. Respir. J..

[2] Lyons O.D., Bradley T.D. (2015). Heart Failure and Sleep Apnea. Can. J. Cardiol..

[3] Harding S.M. (2000). Complications and Consequences of Obstructive Sleep Apnea. Curr. Opin. Pulm. Med..

[4] Benjafield A.V., Ayas N.T., Eastwood P.R., Heinzer R., Ip M.S.M., Morrell M.J., Nunez C.M., Patel S.R., Penzel T., Pépin J.-L. (2019). Estimation of the Global Prevalence and Burden of Obstructive Sleep Apnoea: A Literature-Based Analysis. Lancet. Respir. Med..

[5] Young T., Palta M., Dempsey J., Peppard P.E., Nieto F.J., Hla K.M. (2009). Burden of Sleep Apnea: Rationale, Design, and Major Findings of the Wisconsin Sleep Cohort Study. WMJ.

[6] Peppard P.E., Young T., Barnet J.H., Palta M., Hagen E.W., Hla K.M. (2013). Increased Prevalence of Sleep-Disordered Breathing in Adults. Am. J. Epidemiol..

[7] Heinzer R., Vat S., Marques-Vidal P., Marti-Soler H., Andries D., Tobback N., Mooser V., Preisig M., Malhotra A., Waeber G. (2015). Prevalence of Sleep-Disordered Breathing in the General Population: The HypnoLaus Study. Lancet. Respir. Med..

[8] Dickinson K.M., Collaco J.M. (2021). Cystic Fibrosis. Pediatr. Rev..

[9] Polverino E., Goeminne P.C., McDonnell M.J., Aliberti S., Marshall S.E., Loebinger M.R., Murris M., Cantón R., Torres A., Dimakou K. (2017). European Respiratory Society Guidelines for the Management of Adult Bronchiectasis. Eur. Respir. J..

[10] Borekci S., Sekibag Y., Harbiyeli D.O., Musellim B. (2021). The Frequency of Obstructive Sleep Apnea in Patients with Non-Cystic Fibrosis Bronchiectasis. Turkish Thorac. J..

[11] Júnior N.S.F., Urbano J.J., Santos I.R., Silva A.S., Perez E.A., Souza H., Nascimento O.A., Jardim J.R., Insalaco G., Oliveira L.V.F. (2017). Evaluation of Obstructive Sleep Apnea in Non-Cystic Fibrosis Bronchiectasis: A Cross-Sectional Study. PLoS ONE.

[12] Mori K., Tabusadani M., Yamane K., Takao S., Kuroyama Y., Matsumura Y., Ono K., Kawahara K., Omatsu S., Fujiwara K. (2021). Effects of Pain on Depression, Sleep, Exercise Tolerance, and Quality of Life in Patients with Nontuberculous Mycobacterial Pulmonary Disease. Medicine.

[13] Shakkottai A., Nasr S.Z., Hassan F., Irani S., O’Brien L.M., Chervin R.D. (2020). Sleep-Disordered Breathing in Cystic Fibrosis. Sleep Med..

[14] Barbosa R.R.B., Liberato F.M.G., de Freitas Coelho P., Vidal P.D.R., de Carvalho R.B.C.O., Donadio M.V.F. (2020). Sleep-Disordered Breathing and Markers of Morbidity in Children and Adolescents with Cystic Fibrosis. Pediatr. Pulmonol..

[15] Shakkottai A., Irani S., Nasr S.Z., O’Brien L.M., Chervin R.D. (2022). Risk Factors for Obstructive Sleep Apnea in Cystic Fibrosis. Pediatr. Pulmonol..

[16] Lumertz M.S., Pinto L.A. (2019). Sleep-Disordered Breathing in Cystic Fibrosis Pediatric Subjects. Sleep Sci..

[17] Piper A.J., Grunstein R.R. (2011). Obesity Hypoventilation Syndrome: Mechanisms and Management. Am. J. Respir. Crit. Care Med..

[18] Ward S., Chatwin M., Heather S., Simonds A.K. (2005). Randomised Controlled Trial of Non-Invasive Ventilation (NIV) for Nocturnal Hypoventilation in Neuromuscular and Chest Wall Disease Patients with Daytime Normocapnia. Thorax.

[19] McNicholas W.T., Hansson D., Schiza S., Grote L. (2019). Sleep in Chronic Respiratory Disease: COPD and Hypoventilation Disorders. Eur. Respir. Rev. Off. J. Eur. Respir. Soc..

[20] Dempsey J.A., Xie A., Patz D.S., Wang D. (2014). Physiology in Medicine: Obstructive Sleep Apnea Pathogenesis and Treatment—Considerations beyond Airway Anatomy. J. Appl. Physiol..

[21] Malhotra A., Owens R.L. (2010). What Is Central Sleep Apnea?. Respir. Care.

[22] Dempsey J.A., Veasey S.C., Morgan B.J., O’Donnell C.P. (2010). Pathophysiology of Sleep Apnea. Physiol. Rev..

[23] Sánchez-de-la-Torre M., Campos-Rodriguez F., Barbé F. (2013). Obstructive Sleep Apnoea and Cardiovascular Disease. Lancet. Respir. Med..

[24] Eckert D.J., White D.P., Jordan A.S., Malhotra A., Wellman A. (2013). Defining Phenotypic Causes of Obstructive Sleep Apnea. Identification of Novel Therapeutic Targets. Am. J. Respir. Crit. Care Med..

[25] Wellman A., Eckert D.J., Jordan A.S., Edwards B.A., Passaglia C.L., Jackson A.C., Gautam S., Owens R.L., Malhotra A., White D.P. (2011). A Method for Measuring and Modeling the Physiological Traits Causing Obstructive Sleep Apnea. J. Appl. Physiol..

[26] Wellman A., Edwards B.A., Sands S.A., Owens R.L., Nemati S., Butler J., Passaglia C.L., Jackson A.C., Malhotra A., White D.P. (2013). A Simplified Method for Determining Phenotypic Traits in Patients with Obstructive Sleep Apnea. J. Appl. Physiol..

[27] Younes M. (2003). Contributions of Upper Airway Mechanics and Control Mechanisms to Severity of Obstructive Apnea. Am. J. Respir. Crit. Care Med..

[28] Vicente E., Marin J.M., Carrizo S.J., Osuna C.S., González R., Marin-Oto M., Forner M., Vicente P., Cubero P., Gil A.V. (2016). Upper Airway and Systemic Inflammation in Obstructive Sleep Apnoea. Eur. Respir. J..

[29] Kheirandish-Gozal L., Gozal D. (2019). Obstructive Sleep Apnea and Inflammation: Proof of Concept Based on Two Illustrative Cytokines. Int. J. Mol. Sci..

[30] Goldbart A.D., Krishna J., Li R.C., Serpero L.D., Gozal D. (2006). Inflammatory Mediators in Exhaled Breath Condensate of Children with Obstructive Sleep Apnea Syndrome. Chest.

[31] Ravimohan S., Kornfeld H., Weissman D., Bisson G.P. (2018). Tuberculosis and Lung Damage: From Epidemiology to Pathophysiology. Eur. Respir. Rev. Off. J. Eur. Respir. Soc..

[32] Chalmers J.D., Chang A.B., Chotirmall S.H., Dhar R., McShane P.J. (2018). Bronchiectasis. Nat. Rev. Dis. Prim..

[33] Fuschillo S., De Felice A., Balzano G. (2008). Mucosal Inflammation in Idiopathic Bronchiectasis: Cellular and Molecular Mechanisms. Eur. Respir. J..

[34] Horner R.L., Hughes S.W., Malhotra A. (2014). State-Dependent and Reflex Drives to the Upper Airway: Basic Physiology with Clinical Implications. J. Appl. Physiol..

[35] Perger E., Jutant E.-M., Redolfi S. (2018). Targeting Volume Overload and Overnight Rostral Fluid Shift: A New Perspective to Treat Sleep Apnea. Sleep Med. Rev..

[36] Redolfi S., Yumino D., Ruttanaumpawan P., Yau B., Su M.-C., Lam J., Bradley T.D. (2009). Relationship between Overnight Rostral Fluid Shift and Obstructive Sleep Apnea in Nonobese Men. Am. J. Respir. Crit. Care Med..

[37] Kim A.M., Keenan B.T., Jackson N., Chan E.L., Staley B., Poptani H., Torigian D.A., Pack A.I., Schwab R.J. (2014). Tongue Fat and Its Relationship to Obstructive Sleep Apnea. Sleep.

[38] Paulsen F.P., Steven P., Tsokos M., Jungmann K., Müller A., Verse T., Pirsig W. (2002). Upper Airway Epithelial Structural Changes in Obstructive Sleep-Disordered Breathing. Am. J. Respir. Crit. Care Med..

[39] Boyd J.H., Petrof B.J., Hamid Q., Fraser R., Kimoff R.J. (2004). Upper Airway Muscle Inflammation and Denervation Changes in Obstructive Sleep Apnea. Am. J. Respir. Crit. Care Med..

[40] McDonnell M.J., O’Toole D., Ward C., Pearson J.P., Lordan J.L., De Soyza A., Loebinger M., Chalmers J.D., Laffey J.G., Rutherford R.M. (2018). A Qualitative Synthesis of Gastro-Oesophageal Reflux in Bronchiectasis: Current Understanding and Future Risk. Respir. Med..

[41] Ledson M.J., Wilson G.E., Tran J., Walshaw M.J. (1998). Tracheal Microaspiration in Adult Cystic Fibrosis. J. R. Soc. Med..

[42] Lee A.S., Lee J.S., He Z., Ryu J.H. (2020). Reflux-Aspiration in Chronic Lung Disease. Ann. Am. Thorac. Soc..

[43] Shukla S.D., Walters E.H., Simpson J.L., Keely S., Wark P.A.B., O’Toole R.F., Hansbro P.M. (2020). Hypoxia-Inducible Factor and Bacterial Infections in Chronic Obstructive Pulmonary Disease. Respirology.

[44] Locke B.W., Lee J.J., Sundar K.M. (2022). OSA and Chronic Respiratory Disease: Mechanisms and Epidemiology. Int. J. Environ. Res. Public Health.

[45] Shteinberg M., Nassrallah N., Jrbashyan J., Uri N., Stein N., Adir Y. (2018). Upper Airway Involvement in Bronchiectasis Is Marked by Early Onset and Allergic Features. ERJ Open Res..

[46] Javaheri S., Kazemi H. (1987). Metabolic Alkalosis and Hypoventilation in Humans. Am. Rev. Respir. Dis..

[47] Khoo M.C., Kronauer R.E., Strohl K.P., Slutsky A.S. (1982). Factors Inducing Periodic Breathing in Humans: A General Model. J. Appl. Physiol..

[48] Ghazanshahi S.D., Khoo M.C. (1997). Estimation of Chemoreflex Loop Gain Using Pseudorandom Binary CO_2_ Stimulation. IEEE Trans. Biomed. Eng..

[49] White D.P. (2005). Pathogenesis of Obstructive and Central Sleep Apnea. Am. J. Respir. Crit. Care Med..

[50] Rosen M.J. (2006). Chronic Cough Due to Bronchiectasis: ACCP Evidence-Based Clinical Practice Guidelines. Chest.

[51] Penketh A.R., Wise A., Mearns M.B., Hodson M.E., Batten J.C. (1987). Cystic Fibrosis in Adolescents and Adults. Thorax.

[52] Turner R.D. (2019). Cough in Pulmonary Tuberculosis: Existing Knowledge and General Insights. Pulm. Pharmacol. Ther..

[53] Horner R.L., Sanford L.D., Pack A.I., Morrison A.R. (1997). Activation of a Distinct Arousal State Immediately after Spontaneous Awakening from Sleep. Brain Res..

[54] Malhotra A., Mesarwi O., Pepin J.-L., Owens R.L. (2020). Endotypes and Phenotypes in Obstructive Sleep Apnea. Curr. Opin. Pulm. Med..

[55] Hlavac M.C., Catcheside P.G., McDonald R., Eckert D.J., Windler S., McEvoy R.D. (2006). Hypoxia Impairs the Arousal Response to External Resistive Loading and Airway Occlusion during Sleep. Sleep.

[56] Júnior N.S.F., Oliveira L.V.F., Perez E.A., De Oliveira E.F., Apostolico N., Pereira N.A., Santos I.D.R.D., Urbano J.J., Souza I.D., Polonio I.B. (2015). Observational Study of Sleep, Respiratory Mechanics and Quality of Life in Patients with Non-Cystic Fibrosis Bronchiectasis: A Protocol Study. BMJ Open.

[57] Radovanovic D., Santus P., Blasi F., Sotgiu G., D’Arcangelo F., Simonetta E., Contarini M., Franceschi E., Goeminne P.C., Chalmers J.D. (2018). A Comprehensive Approach to Lung Function in Bronchiectasis. Respir. Med..

[58] Gao Y., Guan W., Xu G., Lin Z., Tang Y., Lin Z., Li H., Gao Y., Luo Q., Zhong N. (2014). Sleep Disturbances and Health-Related Quality of Life in Adults with Steady-State Bronchiectasis. PLoS ONE.

[59] Erdem E., Ersu R., Karadag B., Karakoc F., Gokdemir Y., Ay P., Akpinar I.N., Dagli E. (2011). Effect of Night Symptoms and Disease Severity on Subjective Sleep Quality in Children with Non-Cystic-Fibrosis Bronchiectasis. Pediatr. Pulmonol..

[60] Yang X., Tang X., Cao Y., Dong L., Wang Y., Zhang J., Cao J. (2020). The Bronchiectasis in COPD-OSA Overlap Syndrome Patients. Int. J. Chron. Obstruct. Pulmon. Dis..

[61] Oktem S., Karadag B., Erdem E., Gokdemir Y., Karakoc F., Dagli E., Ersu R. (2013). Sleep Disordered Breathing in Patients with Primary Ciliary Dyskinesia. Pediatr. Pulmonol..

[62] Santamaria F., Esposito M., Montella S., Cantone E., Mollica C., De Stefano S., Mirra V., Carotenuto M. (2014). Sleep Disordered Breathing and Airway Disease in Primary Ciliary Dyskinesia. Respirology.

[63] Reiter J., Gileles-Hillel A., Cohen-Cymberknoh M., Rosen D., Kerem E., Gozal D., Forno E. (2020). Sleep Disorders in Cystic Fibrosis: A Systematic Review and Meta-Analysis. Sleep Med. Rev..

[64] Perin C., Fagondes S.C., Casarotto F.C., Pinotti A.F.F., Barreto S.S.M., Dalcin P.d.T.R. (2012). Sleep Findings and Predictors of Sleep Desaturation in Adult Cystic Fibrosis Patients. Sleep Breath..

[65] Shakkottai A., O’Brien L.M., Nasr S.Z., Chervin R.D. (2018). Sleep Disturbances and Their Impact in Pediatric Cystic Fibrosis. Sleep Med. Rev..

[66] Spicuzza L., Sciuto C., Leonardi S., La Rosa M. (2012). Early Occurrence of Obstructive Sleep Apnea in Infants and Children with Cystic Fibrosis. Arch. Pediatr. Adolesc. Med..

[67] Villa M.P., Pagani J., Lucidi V., Palamides S., Ronchetti R. (2001). Nocturnal Oximetry in Infants with Cystic Fibrosis. Arch. Dis. Child..

[68] Reiter J., Breuer O., Cohen-Cymberknoh M., Forno E., Gileles-Hillel A. (2022). Sleep in Children with Cystic Fibrosis: More under the Covers. Pediatr. Pulmonol..

[69] Jagpal S.K., Jobanputra A.M., Ahmed O.H., Santiago T.V., Ramagopal M. (2021). Sleep-Disordered Breathing in Cystic Fibrosis. Pediatr. Pulmonol..

[70] Milross M.A., Piper A.J., Dobbin C.J., Bye P.T.P., Grunstein R.R. (2004). Sleep Disordered Breathing in Cystic Fibrosis. Sleep Med. Rev..

[71] de Sousa L.P., Liberato F.M.G., Vendrusculo F.M., Donadio M.V.F., Barbosa R.R.B. (2021). Obstructive Sleep Apnea in Children and Adolescents with Cystic Fibrosis and Preserved Lung Function or Mild Impairment: A Systematic Review and Meta-Analysis of Prevalence. Sleep Med..

[72] Senaratna C.V., Perret J.L., Lodge C.J., Lowe A.J., Campbell B.E., Matheson M.C., Hamilton G.S., Dharmage S.C. (2017). Prevalence of Obstructive Sleep Apnea in the General Population: A Systematic Review. Sleep Med. Rev..

[73] Milross M.A., Piper A.J., Norman M., Willson G.N., Grunstein R.R., Sullivan C.E., Bye P.T. (2001). Predicting Sleep-Disordered Breathing in Patients with Cystic Fibrosis. Chest.

[74] Welsner M., Dietz-Terjung S., Stehling F., Schulte T., Niehammer U., Gahbiche F.-E., Taube C., Strassburg S., Schoebel C., Weinreich G. (2022). Obstructive Sleep Apnea and Nocturnal Hypoxemia in Adult Patients with Cystic Fibrosis. BMC Pulm. Med..

[75] McKone E.F., Ariti C., Jackson A., Zolin A., Carr S.B., Orenti A., van Rens J.G., Lemonnier L., Macek M.J., Keogh R.H. (2021). Survival Estimates in European Cystic Fibrosis Patients and the Impact of Socioeconomic Factors: A Retrospective Registry Cohort Study. Eur. Respir. J..

[76] Kutney K.A., Sandouk Z., Desimone M., Moheet A. (2021). Obesity in Cystic Fibrosis. J. Clin. Transl. Endocrinol..

[77] Bouka A., Tiede H., Liebich L., Dumitrascu R., Hecker C., Reichenberger F., Mayer K., Seeger W., Schulz R. (2012). Quality of Life in Clinically Stable Adult Cystic Fibrosis Out-Patients: Associations with Daytime Sleepiness and Sleep Quality. Respir. Med..

[78] Veronezi J., Carvalho A.P., Ricachinewsky C., Hoffmann A., Kobayashi D.Y., Piltcher O.B., Abreu e Silva F.A., Martinez D. (2015). Sleep-Disordered Breathing in Patients with Cystic Fibrosis. J. Bras. Pneumol. Publicacao Of. Soc. Bras. Pneumol. Tisilogia.

[79] Ramos R.T.T., Salles C., Gregório P.B., Barros A.T., Santana A., Araújo-Filho J.B., Acosta A.X. (2009). Evaluation of the Upper Airway in Children and Adolescents with Cystic Fibrosis and Obstructive Sleep Apnea Syndrome. Int. J. Pediatr. Otorhinolaryngol..

[80] Katz E.S. (2014). Cystic Fibrosis and Sleep. Clin. Chest Med..

[81] Young A.C., Wilson J.W., Kotsimbos T.C., Naughton M.T. (2008). Randomised Placebo Controlled Trial of Non-Invasive Ventilation for Hypercapnia in Cystic Fibrosis. Thorax.

[82] Wadsworth L.E., Belcher J., Bright-Thomas R.J. (2021). Non-Invasive Ventilation Is Associated with Long-Term Improvements in Lung Function and Gas Exchange in Cystic Fibrosis Adults with Hypercapnic Respiratory Failure. J. Cyst. Fibros. Off. J. Eur. Cyst. Fibros. Soc..

[83] Houben R.M.G.J., Dodd P.J. (2016). The Global Burden of Latent Tuberculosis Infection: A Re-Estimation Using Mathematical Modelling. PLoS Med..

[84] Mesquita E.D.D., Gil-Santana L., Ramalho D., Tonomura E., Silva E.C., Oliveira M.M., Andrade B.B., Kritski A. (2016). Associations between Systemic Inflammation, Mycobacterial Loads in Sputum and Radiological Improvement after Treatment Initiation in Pulmonary TB Patients from Brazil: A Prospective Cohort Study. BMC Infect. Dis..

[85] Kaufmann S.H.E., Dorhoi A. (2013). Inflammation in Tuberculosis: Interactions, Imbalances and Interventions. Curr. Opin. Immunol..

[86] Sharma S.K., Upadhyay V. (2020). Epidemiology, Diagnosis & Treatment of Non-Tuberculous Mycobacterial Diseases. Indian J. Med. Res..

[87] Winthrop K.L., Marras T.K., Adjemian J., Zhang H., Wang P., Zhang Q. (2020). Incidence and Prevalence of Nontuberculous Mycobacterial Lung Disease in a Large U.S. Managed Care Health Plan, 2008–2015. Ann. Am. Thorac. Soc..

[88] Faverio P., Stainer A., Bonaiti G., Zucchetti S.C., Simonetta E., Lapadula G., Marruchella A., Gori A., Blasi F., Codecasa L. (2016). Characterizing Non-Tuberculous Mycobacteria Infection in Bronchiectasis. Int. J. Mol. Sci..

[89] Vadakkan Devassy T., Ps N., Sharma D., Thomas A.M. (2022). Sleep Disorders in Elderly Population Suffering from TB and Respiratory Diseases. Indian J. Tuberc..

[90] Lee T., Tsai M.-J., Chung Y.-C., Huang H.-L., Chang W.-A., Chong I.-W., Huang M.-S. (2014). Association between Sleep Apnea and Tuberculosis—A Nationwide Population-Based Study. Eur. Respir. J..

[91] Patel A.B., Hinni M.L. (2013). Tuberculous Retropharyngeal Abscess Presenting with Symptoms of Obstructive Sleep Apnea. Eur. Arch. Oto-Rhino-Laryngology.

[92] Yilmaz Y.F., Tezer M.S., Titiz A., Özlügedik S., Yalçin F., Ünal A. (2005). Snoring and Obstructive Sleep Apnea Due to Nasopharyngeal Tuberculosis. Gazi Med. J..

[93] Sharma H.S., Kurl D.N., Kamal M.Z. (1998). Tuberculoid Granulomatous Lesion of the Pharynx--Review of the Literature. Auris. Nasus. Larynx.

[94] Sakuma T., Tatsumi K., Kimura H., Honda Y., Kuriyama T. (1996). Sleep Oxygen Desaturation in Late Sequelae of Pulmonary Tuberculosis. Intern. Med..

[95] Matsumura Y., Tabusadani M., Yamane K., Takao S., Kuroyama Y., Mori K., Ono K., Kawahara K., Omatsu S., Furuuchi K. (2022). Prevalence of and Risk Factors for Depressive Symptoms in Non-Tuberculous Mycobacterial Pulmonary Disease. Int. J. Tuberc. Lung Dis..

[96] Bonsignore M.R., McNicholas W.T., Montserrat J.M., Eckel J. (2012). Adipose Tissue in Obesity and Obstructive Sleep Apnoea. Eur. Respir. J..

[97] Chan K.K.Y., Ing A.J., Laks L., Cossa G., Rogers P., Birring S.S. (2010). Chronic Cough in Patients with Sleep-Disordered Breathing. Eur. Respir. J..

[98] Birring S.S., Ing A.J., Chan K., Cossa G., Matos S., Morgan M.D.L., Pavord I.D. (2007). Obstructive Sleep Apnoea: A Cause of Chronic Cough. Cough.

[99] Eltzschig H.K., Carmeliet P. (2011). Hypoxia and Inflammation. N. Engl. J. Med..

[100] Montgomery S.T., Mall M.A., Kicic A., Stick S.M. (2017). Hypoxia and Sterile Inflammation in Cystic Fibrosis Airways: Mechanisms and Potential Therapies. Eur. Respir. J..

[101] Perger E., Baillieul S., Esteve F., Pichon A., Bilo G., Soranna D., Doutreleau S., Savina Y., Ulliel-Roche M., Brugniaux J.V. (2022). Nocturnal Hypoxemia, Blood Pressure, Vascular Status and Chronic Mountain Sickness in the Highest City in the World. Ann. Med..

[102] Perger E., Soranna D., Pengo M., Meriggi P., Lombardi C., Parati G. (2021). Sleep-Disordered Breathing among Hospitalized Patients with COVID-19. Am. J. Respir. Crit. Care Med..

[103] Wang Y., Hu K., Liu K., Li Z., Yang J., Dong Y., Nie M., Chen J., Ruan Y., Kang J. (2015). Obstructive Sleep Apnea Exacerbates Airway Inflammation in Patients with Chronic Obstructive Pulmonary Disease. Sleep Med..

[104] Faraut B., Boudjeltia K.Z., Vanhamme L., Kerkhofs M. (2012). Immune, Inflammatory and Cardiovascular Consequences of Sleep Restriction and Recovery. Sleep Med. Rev..

[105] Besedovsky L., Lange T., Born J. (2012). Sleep and Immune Function. Pflugers Arch..

[106] Allen J.M., Graef D.M., Ehrentraut J.H., Tynes B.L., Crabtree V.M. (2016). Sleep and Pain in Pediatric Illness: A Conceptual Review. CNS Neurosci. Ther..

[107] Kaczmarski P., Karuga F.F., Szmyd B., Sochal M., Białasiewicz P., Strzelecki D., Gabryelska A. (2022). The Role of Inflammation, Hypoxia, and Opioid Receptor Expression in Pain Modulation in Patients Suffering from Obstructive Sleep Apnea. Int. J. Mol. Sci..

[108] Tuleta I., Stöckigt F., Juergens U.R., Pizarro C., Schrickel J.W., Kristiansen G., Nickenig G., Skowasch D. (2016). Intermittent Hypoxia Contributes to the Lung Damage by Increased Oxidative Stress, Inflammation, and Disbalance in Protease/Antiprotease System. Lung.

